# Prospects and good experimental practices for photocatalytic ammonia synthesis

**DOI:** 10.1038/s41467-022-35489-7

**Published:** 2022-12-23

**Authors:** Po-Wei Huang, Marta C. Hatzell

**Affiliations:** 1 School of Chemical & Biomolecular Engineering, Georgia Institute of Technology, Atlanta, GA 30318 USA; 2 School of Mechanical Engineering, Georgia Institute of Technology, Atlanta, GA 30318 USA

**Keywords:** Photocatalysis, Photocatalysis, Photocatalysis

## Abstract

The development of photocatalysts is greatly hindered by false positives or non-reproducible data. Here, The authors describe the current known causes of non-reproducible results in the literature and present solutions to mitigate these false positive results.

## Teaser

Photocatalytic conversion of nitrogen to ammonia is a green alternative for the Haber-Bosch process. The current progress in photocatalytic nitrogen reduction suggests, however, that there exists a large gap in performance before commercial use is viable. One of the major challenges is that no highly active photocatalysts exist. Furthermore, the development of photocatalysts is greatly hindered by false positives or non-reproducible data. Here, we will describe the current known causes of non-reproducible results in the literature and present solutions to mitigate these false positive results. Finally, we highlight the main challenges that remain for photocatalytic nitrogen fixation. We aim to help researchers design more reliable experiments and inspire practical research in developing photocatalytic ammonia synthesis.

Half of the global population today is fed by crops grown with nitrogen-rich synthetic fertilizers^1,2^. Ammonia is the key ingredient of nitrogen-rich synthetic fertilizer, with 88% of annual ammonia production used for fertilizer production^3^. The current industrial production of ammonia is primarily via the Haber–Bosch process, a thermocatalytic method that converts dinitrogen and dihydrogen into ammonia at elevated temperatures, developed by Fritz Haber and Carl Bosch in the early 1900s^1,4^. The discovery of the Haber–Bosch process contributed to a spike in crop yield, which enabled the population boom in the 20th century, making it the backbone of modern society.

However, the Haber–Bosch process is energy-intensive, accounting for 2% of the global energy consumption, and is accompanied by >500 Mt of CO_2_ (about 1.6% of global CO_2_ emission) emitted yearly^4–8^. In addition, the process requires pure hydrogen feedstock as well as high temperature and pressure operating conditions to ensure high efficiency, resulting in current ammonia production being centralized in large plants in developed countries^9^. Highly centralized ammonia production leads to extra transportation costs and additional carbon emissions in the distribution of fertilizer, limiting the accessibility of economical fertilizer in remote and impoverished regions, which suffered the most from starvation^9–11^. Overfertilization, caused by poor management of excess fertilizer, has become a growing concern in the developed world^12^. These reasons greatly motivate the need for developing more sustainable and distributed alternative techniques for next-generation ammonia production.

In response to the need to decarbonize ammonia synthesis, attractive alternatives, such as the photoreduction of nitrogen to ammonia, have emerged (Eq. 1)^4,13^. With this approach, a photocatalyst absorbs light (UV/vis/IR) and generates electrons and holes. These photogenerated charge carriers then activate dinitrogen from the air. Then protonation of the activated nitrogen with water (as a source of hydrogen) allows for the generation of ammonia. This carbon-free method relies only on renewable feedstocks (water and air) and sustainable energy input (light) and can be operated under ambient conditions. The photoreduction of nitrogen can synthesize ammonia at a range of scales, making it possible to produce fertilizer in a decentralized manner and further eliminate transportation costs. This is desirable, and could ultimately improve the accessibility of fertilizer in remote regions ^9,14^.

1 $${{{{\rm{N}}}}}_{2\left(g\right)}+3{{{{\rm{H}}}}_{2}O}_{\left(l\right)}\begin{array}{c}{{{{\rm{hv}}}}}\\ \to \\ 298\;{{{{\rm{K}}}}}\end{array}2{{{{\rm{NH}}}}}_{3\left(g\right)}+1.5{{{{\rm{O}}}}}_{2\left(g\right)}$$

Despite the advantages of the photoreduction of nitrogen, photocatalytic ammonia synthesis has received varying degrees of attention over the last few decades. This is largely due to the low ammonia yield, leading to a long-standing debate in the community about whether this reaction can occur or not^4,15–18^. It was not until recently that there has been rapid growth in this field. This is largely due to the field benefiting from the development of advanced measurement techniques and methods to synthesize new catalysts (Table S1)^7,19^. Despite the rise in interest, most investigations have focused on materials screening and optimization^20^. Many have reported high-activity catalysts, which have later been proved to show no activity toward nitrogen reduction reaction (NRR)^19,21^. This is attributed to a lack of robust measurement and product quantification protocols. To this end, there is a growing number of publications in the field of electrochemical NRR discussing the importance of rigorous measurements and developing standard protocols for the field to follow^7,19,22–24^. Here, we will extend this discussion but will focus our efforts on photocatalytic NRR. We will detail good experimental practices as well as highlight the opportunities in this field for future improvement. By pointing out the possible contamination sources and indicating factors that might lead to false positives, we aim to guide researchers to better achieve reproducible results and promote meaningful progress in this field.

## Sources of contamination in the photocatalytic NRR system

**Fig. 1:**
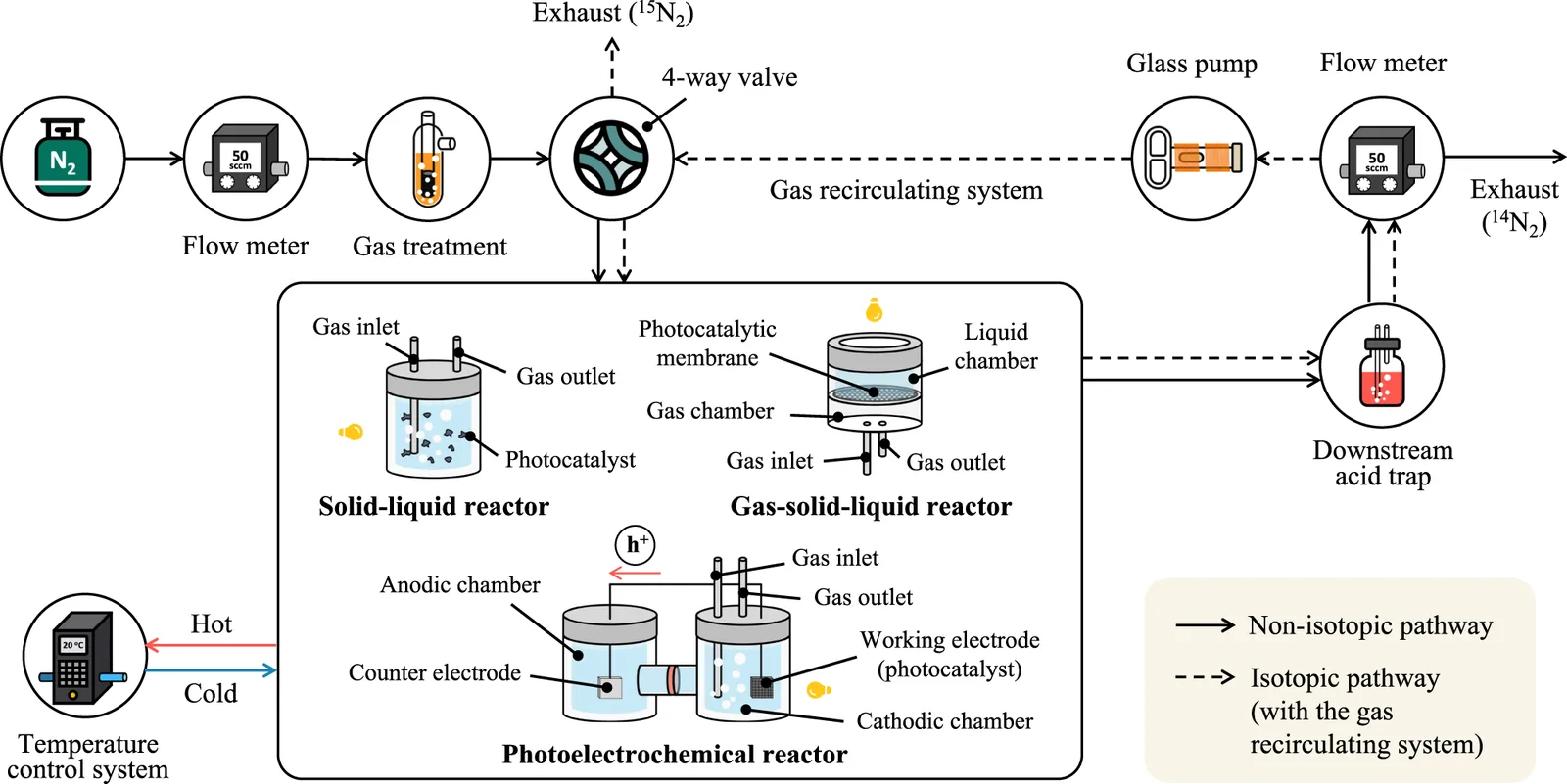
Schematic of the photocatalytic NRR system and different types of photocatalytic reactors. The solid line represents the non-isotopic pathway, and the dashed line represents the isotopic pathway. The first flow meter is to control the gas flow rate and the second flow meter is to check for gas leaks. The detail of the gas recirculating system and the glass pump can be found in refs. ^19, 58^.

The photocatalytic NRR is typically conducted in a heterogeneous suspension system, where a solid powder photocatalyst is directly dispersed in an aqueous solution (water or water with an additive hole scavenger such as methanol or ethanol) with a continuous nitrogen gas flow (Fig. 1). Since the amount of ammonia produced in photocatalytic NRR is small (typically <10 ppm and best case yield around 80 ppm after on hour of reaction^25^), the measure of photocatalysts’ activity can be easily affected by nitrogenous contamination. Therefore, it is imperative to take extra care to eliminate these nitrogenous contaminants and develop robust measurement strategies for this growing field^7^. There are various types of nitrogenous contaminants, spanning from adventitious ammonia to NO_*x*_ species (NO_2_^−^ and NO_3_^−^), and they are ubiquitous. In the following sections, we will highlight the major sources of contamination along with the best practices to suppress contamination.

## Feed gases

Feed gases (^14^N_2_, ^15^N_2_, and Ar) are the most notorious contamination sources in this field, which can contain a significant amount of adventitious ammonia and NO_*x*_ species^19,22,26^. To purify feed gases, the use of deionized water or acidic aqueous solution (e.g., 0.05 M sulfuric acid) traps the impurities in the gas stream. These types of gas treatment are effective, in terms of ammonia removal, but do not remove NO_*x*_. Consideration of adventitious NO_*x*_ species is important as NO_x_ species such as nitrate, and nitrite is thermodynamically more favorable to reduce than dinitrogen^19,22^. Since the solubility of NO_x_ species in deionized water is low^27^ and there has not been a systematic study demonstrating the ability to capture NO_*x*_ species in an acidic solution, we suggest using reduced copper catalyst^19,28^ (for non-aqueous systems) or KMnO_4_ alkaline solution^22^ (for aqueous systems) to eliminate the nitrogenous contaminants in the gas streams.

## Experimental setup

Choosing the proper experimental equipment and materials is essential for conducting rigorous NRR since it has been reported some materials experience degradation throughout the experiment and introduce excess nitrogenous contaminants into the setup^7^. Although the degradation of experimental components (e.g., membrane^29,30^) is a well-known source of contamination in the electrochemical NRR field, there is relatively little discussion on this aspect in the photocatalytic NRR field. Accordingly, the general guideline, for selecting materials (e.g., reactor, tubing, and O-ring) in a photocatalytic NRR setup, is to replace all the components with nitrogen-free materials^7,29^. Such as changing the commonly used nitrile rubber O-ring to the fluoroelastomer O-ring can help with avoiding additional nitrogenous contaminants, but a more systematic study on material contamination in photocatalytic NRR is required.

Furthermore, a strict cleaning procedure for the equipment is required because nitrogenous contaminants can freely accumulate on most surfaces. Based on our own experience, rinsing all the equipment, including pipettes, plastic tubes, glassware, etc., with fresh deionized water between and before use can efficiently control adventitious ammonia. This cleaning process must be taken seriously. For example, the cuvette, which is used for UV–vis measurement, can contain a detectable amount of ammonia, resulting in an overestimation of the produced ammonia for colorimetric techniques. Regarding the suppression of NO_*x*_ contaminants in the setup, washing with alkaline solutions has been reported to be an efficient method^22^. In summary, careful cleaning of all system components that will be in contact with the liquid sample must be completed prior to each experiment.

In addition, the type of water used in the experiments also requires extra attention^31^. Tap water should not be used for NRR experiments as it contains a non-negligible amount of adventitious ammonia. The use of fresh redistilled water or fresh ultrapure water is recommended, but considering that the ammonia contamination of water will vary due to the environment, we recommend that each study measure the ammonia concentration of the water they used and attach it to the report.

## Photocatalysts

In addition to the nitrogenous contamination that is present in the system (feed gases and experimental setup), the catalyst itself is also an inevitable source of contamination. Since ammonia and amine derivatives are commonly used as precursors in the synthesis process, both homemade and commercial catalysts suffer from nitrogenous contamination^7,31,32^. When dispersing this powder photocatalyst into deionized water, without extra care, these surface residual compounds can be spontaneously released into the solution and further bias the measurement of produced ammonia^31^. Especially in the case of nitrogen-containing materials (e.g., graphitic carbon nitride), the catalyst contamination problem can be more severe. The as-prepared nitrogen-containing catalysts, without any treatment, contained a huge amount of nitrogenous contaminants^31^. Like graphitic carbon nitride, one of the most popular photocatalysts, which has shown remarkable activity toward photocatalytic NRR. However, due to the use of nitrogenous precursors in the synthesis process, and the fact that it contains a great amount of nitrogen and surface nitrogen-vacancy, measurements of photo-fixed ammonia by graphitic carbon nitride are prone to serious contamination. For instance, metal nitride has been found to decompose during experiments in the field of electrochemical NRR^21^. Therefore, rigorous operating conditions must be used to ensure that genuine ammonia production is measured. Most of the current research only uses water and ethanol to wash out the organic and inorganic residuals on the as-prepared catalysts, but this is insufficient, so efforts have been done to further purify these catalysts in an electrochemical approach^29^. Still, more work is needed to explore how to effectively purify these photocatalysts.

**Fig. 2:**
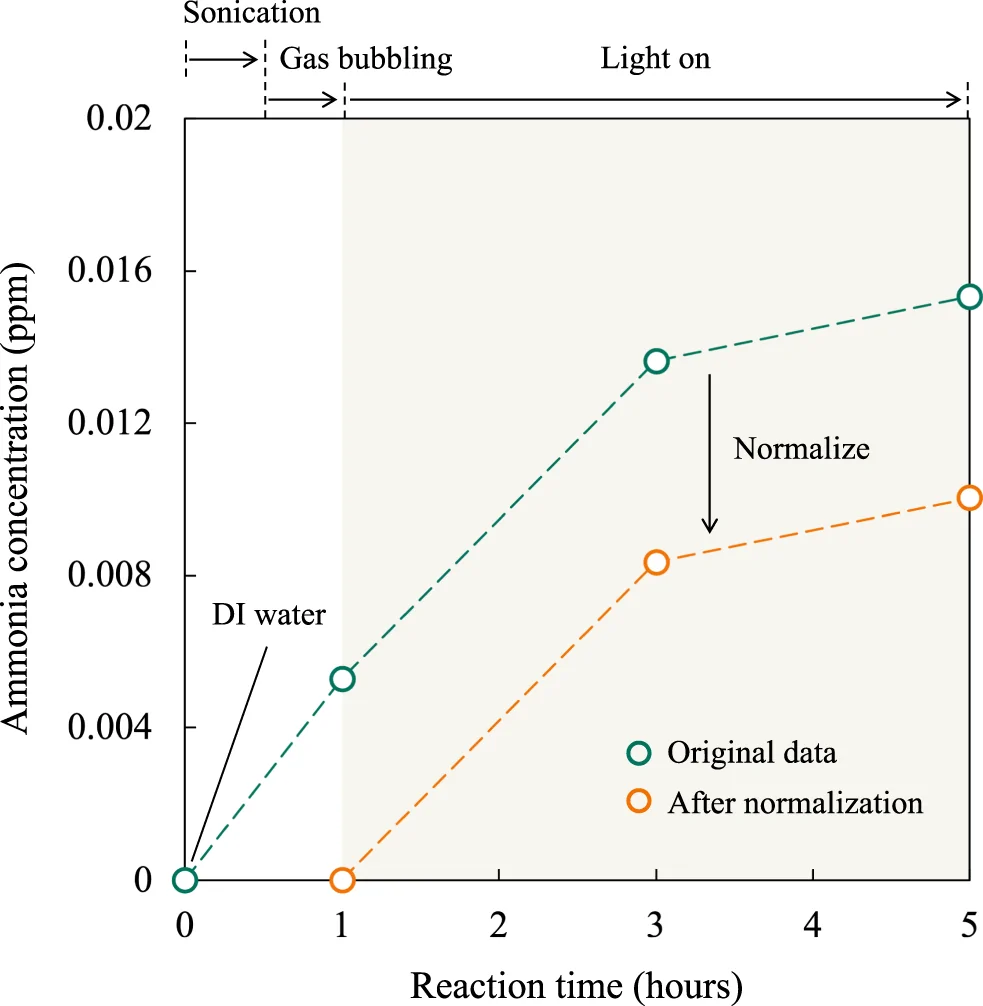
Activity of commercial p25 titanium dioxide. The ammonia concentration versus time (before and after normalization). The experiment is operated under the full spectrum.

Given the operational complexity and the constraints of the system (or material), complete decontamination is not practical. We, therefore, recommend careful cleaning of the setup and catalyst, then quantifying the amount of both ammonia and NO_*x*_ species (NO_2_^−^ and NO_3_^−^) remaining in the system, and reporting their concentration changes over the course of control experiments. In addition, we encourage researchers to report photocatalyst activity in terms of ammonia concentration versus time and attach the unnormalized original data (Fig. 2). This provides a clearer view of the contaminants remaining in the catalyst and helps clarify the source of the final ammonia product.

## Ammonia measurement

To accurately detect ammonia concentration is very important, especially in the low-concentration region, where the quantitative measurement of ammonia below 0.2 ppm is likely error-prone^33^. Typically, non-isotopic ammonia is measured using colorimetric assays (Nessler’s reagent method and indophenol blue method) or ion chromatography, and these measurement methods have similar detection limits in neutral aqueous solutions (ca. 0.01 ppm)^7,34^. However, they will have different degrees of deviation, which can be either positive or negative deviation, under different experimental conditions (composition of the solution, pH, etc.)^31^. Inspired by nitrogenase in nature, many advanced photocatalysts introduced transition metal ions (e.g., iron or molybdenum) to increase activity^35–37^. However, these added metal ions may leach into the solution due to their insufficient photostability. These trace amounts of metal ions in the solution will cause large deviations in the measurement, therefore special attention should be paid to the changes in the pH or ionic composition of the solution before and after the photocatalytic NRR^31^. Measuring the solution composition through ICP-OES, is one way to rule out the impacts of trace metals. In addition, in the field of photocatalysis, alcohols as hole scavengers (e.g., methanol or ethanol) are often used to remove the photo-generated holes, enhance the efficiency of electron–hole separation, and further increase the photocatalytic activity^38^. During photocatalytic experiments, these alcohols will be oxidized to various carbonyl-containing compounds (e.g., formaldehyde, acetaldehyde, and acetone) and interfered with the detection of ammonia, especially in the case of using colorimetric measurements (e.g., Nessler’s reagent method) to detect ammonia^31,39^. Although hole scavengers can enhance photocatalytic activity, studies have shown that most of the activity enhancement may only come from measurement bias caused by the interference effect^39^. To achieve an accurate and rational ammonia measurement, suitable detection methods (e.g., using ion chromatography for samples with hole scavenger) should be selected according to the experimental conditions to avoid interference effect, and the same composition of solution as the experiment should be used when making the calibration curve. Furthermore, it is suggested to use different quantitative methods to ensure the results are consistent ^33^.

It is also worth noting that using a calibration curve made with a large concentration range (e.g., from 0 to 10 ppm) to measure a low-concentration sample (e.g., 0.05 ppm), will also lead to measurement errors. For this reason, a calibration curve made with a proper concentration interval is required for precise ammonia detection. We recommend that the upper limit of the concentration be no more than one order of magnitude above the sample concentration ^31,35–37^.

As for the isotopic ammonia measurement, the use of nuclear magnetic resonance (NMR) to detect isotopic ammonia (^15^NH_3_) has been deemed the most reliable way to measure the activity of photocatalysts^19^. By replacing the feed gas from nitrogen (^14^N_2_) with isotope-labeled nitrogen (^15^N_2_), isotopic ammonia can be obtained during photocatalytic NRR. The isotopic ammonia and non-isotopic ammonia show different characteristic peaks (doublet and triplet peaks) under the measurement of ^1^H NMR, so the produced ammonia can be distinguished from adventitious ammonia. However, the commercial isotope-labeled nitrogen contains a great amount of contamination, including ^15^NH_3_, ^15^NO_2_^−^, and ^15^NO_3_^−^^26^. Therefore, only reporting the isotopically labeled control experiment is insufficient to demonstrate the catalyst’s activity. Future research needs to focus more on quantitative isotope-labeling control experiments with carefully purified ^15^N_2_. Recently, methods have been proposed to effectively purify commercial isotope-labeled nitrogen and conduct appropriate quantitative isotopically labeled control experiments ^19,22^.

## Reproducibility

Ensuring the reproducibility of experiments is a fundamental requirement for advancing the field. To avoid reporting non-reproducible ammonia, strict and well-documented cleaning procedures, measurement methods, and control experiments (dark, Ar, and quantitative NMR) are necessary. Conducting the dark control experiment (bubble photocatalyst with nitrogen gas without illumination) can show the remaining ammonia impurity in the system, and the ammonia measured in the argon control experiment (bubble photocatalyst with argon gas under illumination) can indicate the NO_x_ impurities in the setup. The influence of adventitious ammonia can be well avoided by comparing the experimental results with the control experiments and further identifying the genuine photocatalyst activity. Works have been done to develop rigorous protocols for NRR^7,19,22,31^. The chief conclusions from these protocols are that (i) the reported result must come from multiple testing (>3 times), and the original data must be attached, (ii) quantitative isotopically labeled control experiments must be conducted, (iii) the yield of quantitative isotope-labeling control experiments must be consistent with the experimental results, providing confirmation of genuine photocatalyst activity and ensuring reproducible performance.

**Fig. 3:**
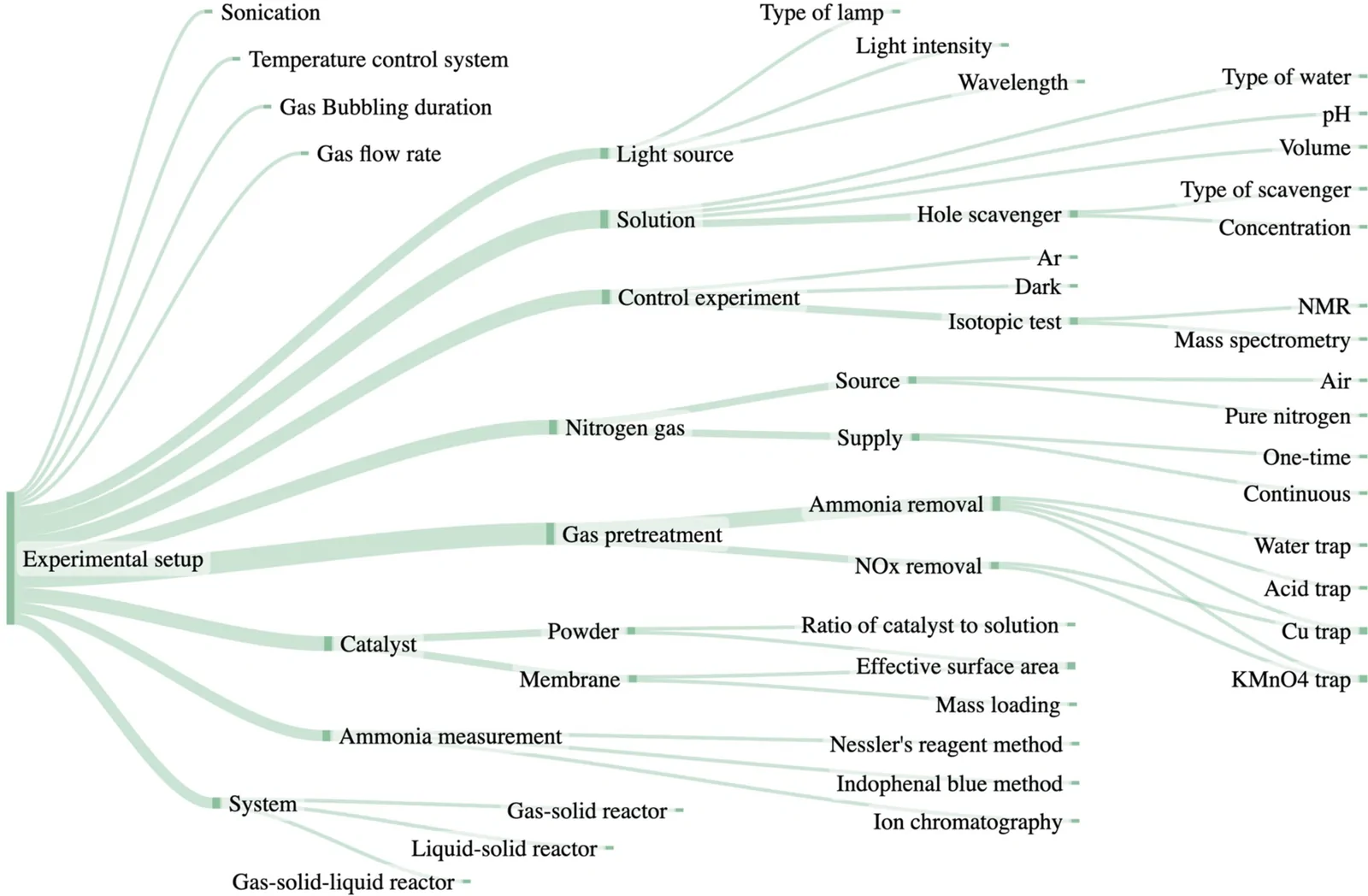
Unstandardized experimental parameters for photocatalytic NRR. Here we detail the parameters that may affect the photocatalytic ammonia yield. These parameters vary in different photocatalytic NRR studies..

Apart from the above discussions, there is a lack of consistency in operating conditions in the field of photocatalytic ammonia synthesis. We listed all the parameters that might affect the performance (Fig. 3). Typically, the reported photocatalysts were tested at a range of temperatures, gas flow rates, concentrations of hole scavengers, light sources, etc., making it difficult to reproduce others’ work and almost impossible to objectively evaluate the performance of different photocatalysts. For example, in a photocatalytic system, the temperature of the solution will change under illumination. Especially for a long duration of testing, if there is no temperature control system (water circulating device), the temperature of the solution can easily rise above 40 °C, and this will lead to higher ammonia concentration in the solution since the higher temperature will favor both the activation of dinitrogen and the desorption of reduced ammonia from the surface of photocatalyst to the solution. However, the importance of the temperature control system has received little attention. Thus, it is essential to apply a temperature control system to a photocatalytic experimental setup and report the temperature changes throughout the experiment. Another example is the unstandardized gas flow rates, which can vary from 20 to 300 sccm in the literature^37,40–42^. Lots of advanced photocatalysts use oxygen vacancies to increase performance by aiding the activation of dinitrogen and suppressing electron-hole recombination^43–46^. However, the change in the flow rate of the gas stream might affect the availability of the surface oxygen vacancy, leading to a different photocatalytic yield and stability ^43,47,48^.

In addition to the inconsistency in operating conditions, the field now requires standardized and rigorous evaluation criteria for the performance of photocatalysts. Taking the photocatalyst activity as an example, the ammonia yield (μmol g_cat_^−1^ h^−1^) is widely present in the literature. In the research of composite catalytic materials, some improbable ammonia yields, however, have been reported when only the weight of the catalyst is considered, but other parts of the catalyst (co-catalyst, photosensitizer, substrate material, etc.) are ignored. Therefore, we strongly recommend reporting detailed parameters (the total mass of the catalyst, effective surface area, etc.) when evaluating photocatalyst performance, which can help avoid overestimated (or underestimated) photocatalytic performances^49^. Solar-to-ammonia (STA) efficiency, though, is one of the most important indicators to commercialize photocatalytic ammonia synthesis technology, which has been overlooked when reporting the performance of photocatalysts (Table S1)^49^. From STA efficiency, we can simultaneously evaluate the light utilization and catalytic efficiency of the material. The STA efficiency is required to be greater than 0.1%, to achieve the potential use of photo-fertilizers to replace conventional fertilizers prepared by the Haber-Bosch process^4^. We, therefore, encourage STA efficiency to be reported in future works.

In summary, in addition to developing a rigorous measuring procedure, a standard testing protocol, in terms of experimental setups, parameters, and evaluation criteria, is also crucial to the development of this field. Since there is not yet a systematic discussion on the effect of these parameters on performance, we encourage scholars to report the experimental parameters as detailed as possible, which is conducive to reproducing the experiment and avoiding irrational comparisons in photocatalytic ammonia production. It is also crucial to explore the impact of various operating variables on the photocatalytic NRR, which can provide further insight into the reaction mechanism.

## Summary and outlook

The production of ammonia through a photocatalytic route is a potential way to produce decarbonized and distributed ammonia. This field is currently in its infancy but is receiving great attention. Various novel photocatalysts and techniques have been developed and applied to boost photocatalytic NRR yield. For instance, metal doping or surface defects have been employed to improve nitrogen adsorption and activation^38,50^. Besides, morphology control or heterojunction construction seems a feasible means to increase electron-hole separation efficiency^20,51^. In addition to the development of catalysts, advances in the design of reaction systems are also worthy of attention. For example, the gas-membrane-solution reaction interface was used to overcome the mass transfer limitation of dissolved nitrogen gas in the solution^52^, and the electron–hole separation efficiency can be improved by using the photoelectrochemical reactor^38^. Recently, progress has been made in photothermal-assisted photocatalytic NRR. It was found that the electron-hole recombination issue can be effectively suppressed under an elevated-temperature environment (>270 °C), and further promoted the yield of ammonia, leading to an unprecedented STA efficiency (0.24%)^53^. However, there are many great challenges to be overcome before this technology can be commercialized, including exploring the reaction mechanism^43,54,55^, studying the effect of solution composition and additives on the reaction performance^31,42,56^, and clarifying the relationship between surface defects and material activity ^43,45,57^.

We believe that at the current stage, rather than materials screening and optimization, it is crucial to develop methods to accurately measure genuine photo-fixed ammonia, avoiding adventitious ammonia or interference during ammonia measurement. Ion chromatography appears to be the most reliable of the current ammonia assays and can avoid most measurement interferences from metal ions or hole scavengers, however, most cation exchange columns used in ion chromatography are not compatible with alcohol hole scavengers. While other colorimetric methods are difficult to avoid the interference effect during the measurement. Despite quantitative isotopically labeled NMR can effectively avoid adventitious ammonia and measure genuine ammonia, considering the high cost, the accessibility of the equipment, and the isotopic ammonia contamination appearing in commercial isotope-labeled nitrogen, it is not a sustainable method for photocatalytic NRR. Therefore, a more facile and reliable method for measuring low concentrations of ammonia is imperative. Meanwhile, all current ammonia assays require breaking the closed system for sampling and measurement. Such a process not only increases the risk of contamination of samples with adventitious ammonia but also limits the data points that can be taken per experiment. Ultimately, an in-line in-operando ammonia measuring method is necessary, which will not only help overcome the aforementioned problems but will also contribute to the study of the photocatalytic NRR mechanism.

In terms of material development, we hope more rigorous results can be reported, rather than non-reproducible data which may mislead the field. To this end, we point out the currently known causes of non-reproducible data and the best solutions at this stage, including possible sources of contamination and proper cleaning methods, reasons for measurement deviations, and the use of appropriate control experiments to demonstrate genuine photocatalyst activity. Due to the low yield of current photocatalytic NRR, we recommend that all experiments (solid–liquid photocatalytic NRR, photoelectrochemical reaction, and photothermal-assisted photocatalytic NRR, etc.) follow this standard to ensure reproducibility. In addition to the known sources of contamination, more efforts must be devoted to exploring the photostability of catalysts and materials. Most photocatalytic experiments are carried out under UV or full-spectrum light sources, this might lead to the degradation of catalysts and materials under illumination, resulting in ammonia contamination or ammonia measurement interference. Further, from the perspective of experimental practice, systematically understanding the influence of various experimental parameters on the reaction, and establishing a standardized testing protocol and rigor evaluation criteria, are the directions that are currently imperative in this field. We believe that if these major limitations can be addressed, they will further advance the development of photocatalytic ammonia synthesis and move forward toward the goal of sustainable food production.

## Supplementary information

Supplementary Information
