# Supplementary material for: Prospects and good experimental practices for photocatalytic ammonia synthesis
Source: Nat Commun. 2022 Dec 23;13:7908. doi: 10.1038/s41467-022-35489-7 (PMC9789054; doi:10.1038/s41467-022-35489-7)
Supplement: Supplementary file 1 — Supplementary Information [file 41467_2022_35489_MOESM1_ESM.pdf]

SUPPLEMENTARY INFORMATION

Prospects and Good Experimental Practices for Photocatalytic Ammonia Synthesis

Po-Wei Huang [a] *and Marta C. Hatzell*[a, b, *]

[a] School of Chemical & Biomolecular Engineering, Georgia Institute of Technology, Atlanta, GA 30318, USA

[b] School of Mechanical Engineering, Georgia Institute of Technology, Atlanta, GA 30318, USA

Corresponding authors: marta.hatzell@me.gatech.edu

Supplementary Table 1. Summary of photocatalytic NRR

Year	Reaction system	Catalyst	N-based material	Light source	Hole scavenger	Temperature control	Yield rate (μmol g^{-1} h^{-1})	Solor-to-ammonia effciency (STA)	Gas preatreatment	Isotopic test	Ref
2015	Solid-liquid reactor	BiOBr-OV	Y	Full specturm	None	Y (25°C)	223.3	N/A	N	N	1
2017	Solid-liquid reactor	OV-TiO_2	N	UV light	None	N	3.5	0.020%	N	Y	2
2017	Solid-liquid reactor	Ga_2O_3-DBD/g-C_3N_4	Y	Visible light	0.04mM Methanol	N	281	N/A	N	N	3
2018	Solid-liquid reactor	Au/TiO_2-OV	Y	Full specturm	10 vol% Methanol	N	187.1	N/A	N	N	4
2018	Solid-liquid reactor	BP/CN	Y	Visible light	5 vol% Methanol	N	579.16	N/A	N	N	5
2018	Solid-liquid reactor	CNK	Y	Full specturm	100 vol% Methanol	Y (25°C)	3632	N/A	N	Y	6
2019	Solid-liquid reactor	Cu/TiO_2-OV	Y	Full specturm	None	Y (25°C)	78.9	N/A	N	Y	7
2019	Solid-liquid reactor	In_2O_3/In_2S_3	Y	Full specturm	None	Y (15°C)	40.04	N/A	N	N	8
2020	Solid-liquid reactor	TiO_2-OV	N	Full specturm	10 vol% Methanol	Y (25°C)	324.86	N/A	N	Y	9
2020	Solid-liquid reactor	$SrTiO_3$	N	Full specturm	10 vol% Ethanol	N	109.15	N/A	Y	Y	10
2020	Solid-liquid reactor	Bi_5O_7Br-OV	Y	Visible light	None	N	1272	N/A	N	N	11
2020	Solid-liquid reactor	Cu-ZnAl-LDH	Y	Full specturm	None	Y (25°C)	110	0.014%	N	Y	12

Supplementary Table 1. Summary of photocatalytic NRR

Year	Reaction system	Catalyst	N-based material	Light source	Hole scavenger	Temperature control	Yield rate (μmol g^{-1} h^{-1})	Solor-to-ammonia effciency (STA)	Gas preatreatment	Isotopic test	Ref
2020	Solid-liquid reactor	BP/CdS	Y	Visible light	1 vol% Methanol	N	240.17	N/A	N	N	13
2020	Solid-liquid reactor	Au/HCNS-NV	Y	Visible light	20 vol% Methanol	N	783.4	0.032%	N	N	14
2020	Solid-liquid reactor	Ru-SV-CoS/CN	Y	Full specturm	10 vol% Methanol	N	438	0.042%	N	Y	15
2020	Solid-liquid reactor	WS_2@TiO_2	N	Full specturm	0.01M Na_2SO_3	N	1390	N/A	N	N	16
2020	Solid-liquid reactor	Fe-BiOBr	Y	Visible light	None	Y (25°C)	382.68	N/A	N	Y	17
2020	Solid-liquid reactor	Fe-$MoTe_2$	N	Visible light	None	Y (25°C)	129.08	N/A	N	Y	18
2021	Solid-liquid reactor	Au/Mo-$W_{18}O_{49}$	N	Full specturm	20 vol% Methanol	N	399.24	N/A	N	N	19
2021	Solid-liquid reactor	Al-PMOF(Fe)	Y	Visible light	20 vol% Methanol	N	7.47	N/A	Y	Y	20
2021	Solid-liquid reactor	Cu_2O-LDH	N	Visible light	None	Y (25°C)	30.31	N/A	N	Y	21
2021	Solid-liquid reactor	WC-Co/NGC	Y	Full specturm	1mM Na_2SO_3	N	157	N/A	N	Y	22
2021	Gas-solid-liquid reactor	Au@MOF/PTFE	Y	Visible light	None	Y (25°C)	360	N/A	N	Y	23
2021	Solid-liquid reactor	$Bi_2Sn_2O_7$-QD	Y	Full specturm	None	Y (25°C)	334.8	N/A	N	Y	24

Supplementary Table 1. Summary of photocatalytic NRR

Year	Reaction system	Catalyst	N-based material	Light source	Hole scavenger	Temperature control	Yield rate (μmol g^{-1} h^{-1})	Solor-to-ammonia effciency (STA)	Gas preatreatment	Isotopic test	Ref
2021	Solid-liquid reactor	OV-C/TiO_2	N	Full specturm	10mM Methanol	Y (25°C)	84	N/A	N	Y	25
2021	Solid-liquid reactor	C-TiO_x	N	Visible light	10 vol% Methanol	Y (25°C)	109.3	N/A	N	Y	26
2021	Solid-liquid reactor	BMOF(Sr)-Fe	Y	Full specturm	K_2SO_3	N	780	N/A	Y	Y	27
2021	Solid-liquid reactor	Ni_2P-BP	Y	Visible light	1 vol% Methanol	N	6.14	N/A	N	N	28
2021	Solid-liquid reactor	Mn-WO_3	N	Full specturm	None	N	425	0.019%	Y	Y	29
2021	Solid-liquid reactor	S/Cu	N	Full specturm	None	Y	157	0.023%	Y	Y	30
2021	Solid-liquid reactor	GDY@Fe	Y	Visible light	None	N	1762.35	N/A	Y	Y	31
2022	Solid-liquid reactor	CoO-Co_3O_4/RGO	Y	Full specturm	None	N	89.1	N/A	N	Y	32
2022	Solid-liquid reactor	FeN-CDs/TiO_2@CN	Y	Full specturm	5 wt% Methanol	N	624.33	N/A	N	N	33
2022	Solid-liquid reactor	BVO/SV-ZIS	Y	Visible light	None	Y	80.6	0.012%	N	Y	34
2022	Solid-liquid reactor	Ru-SA/H_xMoO_{3-y}	Y	Visible light	None	Y (25°C)	4000	N/A	N	Y	35

***N-based material**: including nitrogen-containing catalysts and catalysts that use nitrogen-containing materials in the synthesis process.

<u>Color code</u>

Solar-to-ammonia efficiency: >20% (potential use as fuel); >0.1% (potential use as fertilizer); <0.1%
Gas pretreatment: NO_x + ammonia removal and report their concentrations; NO_x + ammonia removal; ammonia removal
Isotopic test: Quantitative isotopic test (match with non-isotopic data); qualitative isotopic test only; no isotopic test

References

1. Li, H., Shang, J., Ai, Z. & Zhang, L. Efficient visible light nitrogen fixation with BiOBr nanosheets of oxygen vacancies on the exposed {001} Facets. *J Am Chem Soc* **137**, 6393–6399 (2015).
2. Hirakawa, H., Hashimoto, M., Shiraishi, Y. & Hirai, T. Photocatalytic Conversion of Nitrogen to Ammonia with Water on Surface Oxygen Vacancies of Titanium Dioxide. *J Am Chem Soc* **139**, 10929–10936 (2017).
3. Cao, S., Zhou, N., Gao, F., Chen, H. & Jiang, F. All-solid-state Z-scheme 3,4-dihydroxybenzaldehyde-functionalized Ga2O3/graphitic carbon nitride photocatalyst with aromatic rings as electron mediators for visible-light photocatalytic nitrogen fixation. *Appl Catal B* **218**, 600–610 (2017).
4. Yang, J. *et al.* High-Efficiency 'working-in-Tandem' Nitrogen Photofixation Achieved by Assembling Plasmonic Gold Nanocrystals on Ultrathin Titania Nanosheets. *J Am Chem Soc* **140**, 8497–8508 (2018).
5. Qiu, P., Xu, C., Zhou, N., Chen, H. & Jiang, F. Metal-free black phosphorus nanosheets-decorated graphitic carbon nitride nanosheets with C[sbnd]P bonds for excellent photocatalytic nitrogen fixation. *Appl Catal B* **221**, 27–35 (2018).
6. Li, X., Sun, X., Zhang, L., Sun, S. & Wang, W. Efficient photocatalytic fixation of N2 by KOH-treated g-C3N4. *J Mater Chem A Mater* **6**, 3005–3011 (2018).
7. Zhao, Y. *et al.* Tuning Oxygen Vacancies in Ultrathin TiO 2 Nanosheets to Boost Photocatalytic Nitrogen Fixation up to 700 nm. *Advanced Materials* **31**, (2019).
8. Xu, H. *et al.* Fabrication of In2O3/In2S3 microsphere heterostructures for efficient and stable photocatalytic nitrogen fixation. *Appl Catal B* **257**, (2019).
9. Zhang, G., Yang, X., He, C., Zhang, P. & Mi, H. Constructing a tunable defect structure in TiO2 for photocatalytic nitrogen fixation. *J Mater Chem A Mater* **8**, 334–341 (2020).
10. Huang, B. *et al.* Boosting the photocatalytic activity of mesoporous SrTiO3for nitrogen fixation through multiple defects and strain engineering. *J Mater Chem A Mater* **8**, 22251–22256 (2020).
11. Li, P. *et al.* Visible-Light-Driven Nitrogen Fixation Catalyzed by Bi5O7Br Nanostructures: Enhanced Performance by Oxygen Vacancies. *J Am Chem Soc* **142**, 12430–12439 (2020).
12. Zhang, S. *et al.* Efficient Photocatalytic Nitrogen Fixation over Cuδ+-Modified Defective ZnAl-Layered Double Hydroxide Nanosheets. *Adv Energy Mater* **10**, (2020).
13. Shen, Z. K. *et al.* Few-Layer Black Phosphorus Nanosheets: A Metal-Free Cocatalyst for Photocatalytic Nitrogen Fixation. *ACS Appl Mater Interfaces* **12**, 17343–17352 (2020).
14. Guo, Y. *et al.* Au nanoparticle-embedded, nitrogen-deficient hollow mesoporous carbon nitride spheres for nitrogen photofixation. *J Mater Chem A Mater* **8**, 16218–16231 (2020).

15. Yuan, J., Yi, X., Tang, Y., Liu, M. & Liu, C. Efficient Photocatalytic Nitrogen Fixation: Enhanced Polarization, Activation, and Cleavage by Asymmetrical Electron Donation to N□N Bond. *Adv Funct Mater* **30**, (2020).
16. Shi, L. *et al.* Promoting nitrogen photofixation over a periodic WS2@TiO2 nanoporous film. *J Mater Chem A Mater* **8**, 1059–1065 (2020).
17. Liu, Y., Hu, Z. & Yu, J. C. Fe enhanced visible-light-driven nitrogen fixation on biobr nanosheets. *Chemistry of Materials* **32**, 1488–1494 (2020).
18. Li, H. *et al.* The in-built bionic 'moFe cofactor' in Fe-doped two-dimensional MoTe2nanosheets for boosting the photocatalytic nitrogen reduction performance. *J Mater Chem A Mater* **8**, 13038–13048 (2020).
19. Qiu, P. *et al.* Plasmonic gold nanocrystals simulated efficient photocatalytic nitrogen fixation over Mo doped W18O49nanowires. *J Mater Chem A Mater* **9**, 14459–14465 (2021).
20. Shang, S. *et al.* Atomically Dispersed Iron Metal Site in a Porphyrin-Based Metal-Organic Framework for Photocatalytic Nitrogen Fixation. *ACS Nano* **15**, 9670–9678 (2021).
21. Zhang, S. *et al.* Sub-3 nm Ultrafine Cu2O for Visible Light Driven Nitrogen Fixation. *Angewandte Chemie - International Edition* **60**, 2554–2560 (2021).
22. Wang, L. *et al.* WC and cobalt nanoparticles embedded in nitrogen-doped carbon 3D nanocage derived from H3PW12O40@ZIF-67 for photocatalytic nitrogen fixation. *J Mater Chem A Mater* **9**, 2912–2918 (2021).
23. Chen, L. W. *et al.* Metal-Organic Framework Membranes Encapsulating Gold Nanoparticles for Direct Plasmonic Photocatalytic Nitrogen Fixation. *J Am Chem Soc* **143**, 5727–5736 (2021).
24. Zhang, Y. *et al.* Oxygen vacancies in Bi2Sn2O7 quantum dots to trigger efficient photocatalytic nitrogen reduction. *Appl Catal B* **299**, (2021).
25. Qian, J. *et al.* Photocatalytic Nitrogen Reduction by Ti3C2 MXene Derived Oxygen Vacancy-Rich C/TiO2. *Adv Sustain Syst* **5**, (2021).
26. Han, Q. *et al.* Rational Design of High-Concentration Ti3+ in Porous Carbon-Doped TiO2 Nanosheets for Efficient Photocatalytic Ammonia Synthesis. *Advanced Materials* **33**, (2021).
27. Zhao, Z. *et al.* Boosting Nitrogen Activation via Bimetallic Organic Frameworks for Photocatalytic Ammonia Synthesis. *ACS Catal* **11**, 9986–9995 (2021).
28. Shen, Z. K. *et al.* Identifying the role of interface chemical bonds in activating charge transfer for enhanced photocatalytic nitrogen fixation of Ni2P-black phosphorus photocatalysts. *Appl Catal B* **295**, (2021).
29. Zhang, Y. *et al.* Dual-Metal Sites Boosting Polarization of Nitrogen Molecules for Efficient Nitrogen Photofixation. *Advanced Science* **8**, (2021).
30. Xin, Y. *et al.* Atomic-level insights into the activation of nitrogen via hydrogen-bond interaction toward nitrogen photofixation. *Chem* **7**, 2118–2136 (2021).

31. Fang, Y., Xue, Y., Hui, L., Yu, H. & Li, Y. Graphdiyne@Janus Magnetite for Photocatalytic Nitrogen Fixation. *Angewandte Chemie - International Edition* **60**, 3170–3174 (2021).
32. Lu, H. *et al.* All room-temperature synthesis, N2 photofixation and reactivation over 2D cobalt oxides. *Appl Catal B* **304**, (2022).
33. Li, K. *et al.* Fe-carbon dots enhance the photocatalytic nitrogen fixation activity of TiO2@CN heterojunction. *Chemical Engineering Journal* **429**, (2022).
34. Zhang, G. *et al.* S vacancies act as a bridge to promote electron injection from Z-scheme heterojunction to nitrogen molecule for photocatalytic ammonia synthesis. *Chemical Engineering Journal* **433**, (2022).
35. Yin, H. *et al.* Dual Active Centers Bridged by Oxygen Vacancies of Ruthenium Single-Atom Hybrids Supported on Molybdenum Oxide for Photocatalytic Ammonia Synthesis. *Angewandte Chemie - International Edition* **61**, (2022).
